# Intake of herring oils containing cetoleic acid resulted in a lower serum cholesterol concentration in male obese Zucker *fa/fa* rats

**DOI:** 10.1017/S0007114526106655

**Published:** 2026-06-28

**Authors:** Helle Oldernes, Andrea Hansen, Svein Are Mjøs, Eirik Søfteland, Oddrun Anita Gudbrandsen

**Affiliations:** 1 Dietary Protein Research Group, Centre for Nutrition, Department of Clinical Medicine, University of Bergen, Bergen 5021, Norway; 2 Department of Chemistry, University of Bergen, Bergen 5020, Norway; 3 Department of Medicine, Haukeland University Hospital, Bergen, Norway

**Keywords:** Cetoleic acid, *n*-11 MUFA, Herring oil, Cholesterol, Lipogenesis

## Abstract

A high serum total cholesterol (TC) concentration is a major risk factor for CVD, and lifestyle modifications including a healthy diet are among the first-line strategies for lowering cholesterol concentration and reducing CVD risk. Several studies in rodents have demonstrated a lower circulating TC concentration after intake of cetoleic acid (CA, C22:1*n*-11). The primary aim was to investigate the effect of consuming herring oil (HERO) containing CA or a CA concentrate (CECO) on the circulating TC concentration in obese hypercholesterolaemic rats. Secondary aims included investigating effects of CA on a selection of hepatic enzymes and receptors involved in cholesterol metabolism, lipogenesis and VLDL assembly. Thirty male obese Zucker *fa/fa* rats were fed a diet containing either HERO or CECO, containing 0·70 or 1·40 wt% CA, respectively, or a Control diet with soyabean oil for 5 weeks. Data were analysed using one-way ANOVA. The serum TC concentration was lower in the HERO and CECO groups compared with the Control group (17 and 20 percent, respectively). Both the HERO and the CECO diets down-regulated *de novo* lipogenesis, cholesterol esterification and lipidation of VLDL in the liver compared with the Control diet, but did not affect the hepatic cholesterol synthesis, the LDL receptor or the faecal excretion of cholesterol and bile acids. To conclude, rats fed the HERO or CECO diets had a lower serum concentration of TC, probably as a result of down-regulated VLDL secretion in response to lower lipogenesis. This may have relevance for lowering TC in hypercholesterolaemic humans.

CVD is the leading cause of disease burden globally, affecting more than half a billion people annually, and caused more than 20 million deaths in 2021^(1,2)^. A high serum total cholesterol (TC) concentration, and especially a high LDL-cholesterol concentration, is a major risk factor for CVD^(3,4)^. Lifestyle modifications including a healthy diet and use of lipid-lowering drugs are first-line strategies for lowering the cholesterol concentration and reducing the CVD risk^(5)^.

Fish intake has been associated with a lower CVD risk in several studies^(6–8)^, an effect that has been credited to the *n*-3 PUFA EPA (C20:5*n*-3) and DHA (C22:6*n*-3)^(9)^. However, there is controversy regarding the cholesterol-regulating effects of fish oils or concentrates with high EPA and DHA in humans^(10–15)^. Fish oils have diverse and varying fatty acid compositions that are influenced by factors such as the fish species and the diet of the fish, and contain many interesting fatty acids in addition to EPA and DHA. Recently, long-chain MUFA such as cetoleic acid (CA, C22:1*n*-11) has gained increased interest. CA is found in high amounts in oils from certain fish species, such as herring, which feeds on copepods that are rich in wax esters with *n*-11 MUFA.

The available literature on the effects of diets containing fish oils or fish oil concentrates with a high content of CA on cholesterol concentration in rodents was recently summarised and meta-analysed, showing that intake of CA-rich fish oils and concentrates resulted in a lower cholesterol concentration when compared with diets containing either vegetable oils (corn oil, olive oil or soyabean oil) or animal fats (lard or milk fat)^(16)^. Still, the mechanisms behind the lower cholesterol concentration after CA intake in rodents are largely unknown^(16)^.

The Zucker *fa/fa* rat develops obesity due to a defect in the leptin receptor^(17)^ and presents visible obesity already at four weeks of age^(18)^. This rat develops a range of endocrine abnormalities resembling the human metabolic syndrome, including fatty liver and dyslipidaemia, and has a high rate of lipogenesis (*de novo* fatty acid synthesis)^(19)^, elevated synthesis and secretion of VLDL from the liver^(20)^ and a slow removal of chylomicrons from the circulation^(21)^. The obese Zucker *fa/fa* rat is a well-characterised and widely used model for studies of metabolic complications and possible treatments of human obesity^(22)^.

When Zucker Diabetic Sprague–Dawley (ZDSD) rats with overt type 2 diabetes were fed a diet with herring oil, which contain CA, this resulted in lower serum concentrations of TC and HDL-cholesterol, with no effect on LDL-cholesterol and ApoB100 concentrations^(23)^. Further analyses indicated that the HERO diet up-regulated the faecal excretion of bile acids, but did not affect the cholesterol production in the liver, the hepatic *de novo* lipogenesis and secretion of VLDL, or the liver’s capacity to take up cholesterol from the circulation^(23)^. Insulin resistance is shown to induce synthesis of bile acids and to change the composition of bile acids^(24)^ and is also associated with increased VLDL secretion^(25)^. Therefore, in the present study, we wanted to investigate if intake of herring oils would result in a lower serum TC concentration in another rat model, that is, obese Zucker *fa/fa* rats, and whether a higher intake of CA would have a more pronounced effect on TC concentration. The main aim of the present study was to investigate the effect of consuming diets supplemented with either herring oil (HERO, 0·70 g CA/100 g diet) or a CA concentrate (CECO, 1·40 g CA/100 g diet) on the circulating TC concentration in obese hypercholesterolaemic rats without diabetes. The secondary aims were to investigate any effects of CA intake on a selection of hepatic enzymes and receptors involved in cholesterol metabolism, lipogenesis and VLDL assembly with the purpose to try to explain the mechanisms behind any cholesterol-lowering effect of CA intake in these rats. Our hypothesis was that diets supplemented with either a herring oil containing CA or a CA concentrate would result in a lower serum TC concentration in Zucker *fa/fa* rats.

## Methods

### Animals and diets

The design of this study, the housing conditions and the diets are presented in Hansen *et al.* 2025^(26)^. The present paper presents secondary analyses of this intervention study. Thirty male obese Zucker *fa/fa* rats (HsdHlr:ZUCKER-Lepr^fa^) were obtained from Harlan Laboratories. The rats were 4–6 weeks old at arrival and were housed in pairs in individually ventilated cages (IVC-4, 1500U Eurostandard Type IV S cages, Blue Line, Tecniplast) with free access to chow (Teklad Global Diet 2018 S, Inotiv) until they reached 7 weeks of age. The rats were randomly assigned to receive one of the three experimental diets by drawing paper lots from a jar, each group consisting of ten rats. The rats were assigned random numbers that could not be linked to the experimental groups. The experimental semi-purified diets were modified versions of the American Institute of Nutrition’s recommendation for growing laboratory rodents (AIN-93G)^(27)^ and were added 1·6 g methionine/kg diet as recommended by Reeves^(28)^. The diets differed only in their lipid sources, and all diets contained 20 wt% protein (casein), 10 wt% fat and 11·8 wt% sucrose (Table 1). The intervention diets contained refined oil prepared from herring (*Clupea harengus*) residuals (the HERO diet) or a CA concentrate (the CECO diet). The CECO was produced from refined herring oil extracted from herring trimmings; the fatty acids in the herring oil were transesterified to ethyl esters and distilled to separate the fatty acids, and the ethyl esters were enzymatically re-esterified to TAG using glycerol with a maximum of 5 % ethyl esters in the final product. The production process included multiple purification steps to remove unwanted compounds, such as environmental contaminants. The CECO diet was designed with twice as high CA content as the HERO diet, and all diets contained adequate amounts of essential fatty acids according to Reeves *et al.* ^(27)^. The Control diet contained soyabean oil as the only lipid source. All ingredients were purchased from Dyets Inc. except casein (Sigma-Aldrich), HERO (Pelagia AS) and the CA concentrate (Epax Norway AS). The rats had ad libitum access to feed and water. The diets were stored at –15°C, and daily portions were thawed every morning.

**Table 1.** Compositions of the experimental diets Table 1 long description.

Contents (g/100 g diet)	Control diet	HERO diet	CECO diet
Soyabean oil	10·00	6·30	5·75
Herring oil	–	3·70	–
Cetoleic acid concentrate	–	–	4·25
Casein*	21·56	21·56	21·56
Sucrose	9·00	9·00	9·00
Cornstarch	48·23	48·23	48·23
Cellulose	5·00	5·00	5·00
t-Butylhydroquinone	0·0014	0·0014	0·0014
Mineral Mix (AIN-93-MX)^†^	3·50	3·50	3·50
Vitamin Mix (AIN-93-VX)^‡^	1·00	1·00	1·00
Growth and maintenance supplement^§^	1·00	1·00	1·00
L-Methionine	0·16	0·16	0·16
L-Cystine	0·30	0·30	0·30
Choline Bitartrate**	0·25	0·25	0·25

HERO, herring oil; CECO, cetoleic acid concentrate.

* Contains 92·78 % crude protein.

† Contains sucrose (221 g/kg).

‡ Contains sucrose (967 g/kg).

§ Contains vitamin B_12_ (40 mg/kg) and vitamin K1 (25 mg/kg) mixed with sucrose (995 g/kg) and dextrose (5 g/kg).

** Contains 41 % choline.

The rats were gradually habituated to a semi-purified powder diet (AIN-93G) for growing rodents^(27)^ for 4 d before they were moved to large open cages (W115xD67·5xH153, cm Suite Royale XL, Savic®). Each of the three cages consisted of two plastic floors and four plastic platforms with metal ladders and were split into two equal-size halves, housing six rats on the upper half and four rats on the lower half of the cage. The plastic floors and the platforms were strewn with 2HK Nestpak (TAPVEI ® Harjumaa, Estonia OÜ). Both halves of the cage were equipped with identical environmental enrichments: three Fat Rat Huts (red polycarbonate, size 150 mm × 165 mm × 85 mm, Datesand), two cardboard tunnels (Play Tunnel, 100 × 50·8 × 1·25 mm, Datesand), two gnawing blocks (Aspen brick, 100 mm × 20 mm × 20 mm, TAPVEI®), one Rat Corner Home (paper pulp, 23 × 19 × 9·5 cm, Datesand), two 8 g-portions of Bed-R’Nest nesting material (kraft paper, The Andersons, Inc.), one wooden house (pine, 28 × 16 × 18 cm, Trixie Heimtierbedarf GmbH & Co.), one wooden playing roll with a bell (Trixie), one suspension bridge (wooden ladder with rope ladder, play rope and rope ring with wooden block, Trixie), one-two plastic bottles with water (Classic Crystal Deluxe Water Drinking Bottle, Caldex Holdings Ltd) and ceramic bowls containing water or powder feed. The rats were housed in a room with controlled light/dark cycle (dark 20.00–06.00) and temperature 22–23°C.

### Design

The rats were weighed three times per week. To ensure that the rats included in the statistical analyses were non-diabetic, urine was collected on Day 23 for measurement of the glucose/creatinine ratio, and on Day 28, the rats underwent a meal glucose tolerance test as described in detail below. The groups had similar body weight at baseline (mean 301 (sd 9) grams, *P* ANOVA 0·72), and the change in body weight during the intervention period was similar between the groups (mean 76 (sd 11) percent, *P* ANOVA 0·93), as previously presented^(26)^. At the end of the experimental period, that is, after 35 d of feeding the experimental diets, the feed was withdrawn between 06.00 and 07.00, and the rats were fasted for 6 h with free access to drinking water before being euthanised while anaesthetised with isoflurane (Isoba vet, Intervet, Schering-Plough Animal Health) mixed with oxygen. Blood was drawn from the heart and collected in BD Vacutainer SST II Advance gel tubes (Becton, Dickinson and Company) for isolation of serum. Serum aliquots were frozen at −80°C. The liver, the heart, the left kidney and epididymal white adipose tissues (WATepi) from both sides were carefully dissected out and were frozen at −80°C until analysed.

The personnel handling the rats and conducting the analyses were blinded to the rats’ group allocation. The rats were handled and euthanised in random order.

### Testing of glucose tolerance

To ensure that the rats included in the statistical analyses were non-diabetic, all rats underwent a meal glucose tolerance test at Day 28. Rats were housed individually while fasted for 10 h (from 06.30), and glucose was monitored in fasting state and 120 min after receiving an oral dose of 2 g dextrose + sucrose per kg BW. The dextrose + sucrose load was prepared as a modified, semi-purified meal on the basis of AIN-93G^(27)^ with 417 g of dextrose + sucrose per kg diet. The composition of the diet for the meal tolerance test was, presented per kg diet: 70 g soyabean oil, 262 g casein (76 % raw protein; Sigma-Aldrich), 390 g dextrose, 165 g cornstarch, 50 g cellulose, 35 g mineral mix (containing 22·1 % sucrose), 10 g vitamin mix (containing 96·7 % sucrose), 3 g L-cystine, 1·6 g L-methionine, 2·5 g choline bitartrate and 10 g growth and maintenance supplement (containing 99·5 % sucrose); all ingredients except casein were purchased from Dyets Inc. The rats were allowed a maximum of 15 min to finish the meal. The dorsal tail vein was punctuated, and blood glucose was measured using a blood glucose measuring device (Contour Next; Bayer Consumer Care AG). In addition, glucose/creatinine ratio was measured in urine collected on Day 23, using the GLUC2 (Glucose HK) and CREP2 (Creatinine plus ver.2) kits from Roche Diagnostics on the Cobas c111 system (Roche Diagnostics GmbH).

### Determination of fatty acids and cholesterol in the diets

Lipids were extracted from the diets using a mixture of chloroform and methanol as described by Bligh and Dyer^(29)^. For fatty acid analyses, the extracts were added heneicosanoic acid (C21:0) as internal standard, methylated, and the methyl ester samples were quantified as previously described^(30–32)^. For quantifications of cholesterol, the lipid extracts were evaporated to dryness using nitrogen and were re-dissolved in isopropanol before quantification on the Cobas c111 system using the CHOL2 kit (Cholesterol Gen.2) from Roche Diagnostics.

### Analyses in serum

Serum concentrations of TC, LDL-cholesterol, HDL-cholesterol and TAG were quantified on the Cobas c111 system (Roche Diagnostics) using the CHOL2 (Cholesterol Gen.2), LDLC3 (LDL-Cholesterol Gen.3), HDLC4 (HDL-Cholesterol Gen. 4) and TAG (Triglycerides) kits, with the recommended calibrators and controls from Roche Diagnostics. Bile acids were quantified using the Total Bile Acid Assay Kit (Diazyme Laboratories, Inc.) on the Cobas c111 system. NEFA and choline-containing phospholipids in serum were analysed on the Cobas c 111 system using the NEFA FS kit (Diagnostics Systems) and the Phospholipids FS kit (Diagnostics Systems).

Free cholesterol was quantified in serum using the Cholesterol/Cholesteryl Ester Assay Kit (ab65359, from Abcam), and the serum cholesteryl ester (CE) concentration was calculated as the difference between total and free cholesterol. Apo B48 was measured using the MBS753664 Rat Apolipoprotein B48 ELISA Kit (MyBioSource Inc.), and Apo B100 was measured using the MBS723231 Rat Apolipoprotein B100 ELISA Kit (MyBioSource Inc.). Lecithin-cholesterol acyltransferase was measured using the LS-F34827 Rat LCAT ELISA Kit (LifeSpan BioSciences, Inc.). Plates were read on a SpectraMax Plus384 Microplate Reader (Molecular Devices).

All samples for each of the colorimetric assays were analysed on the same day, and all samples were analysed simultaneously on the same 96-well plates for free cholesterol quantification and the ELISA assays. The CV was < 6 % for all colorimetric assays and < 5% for the ELISA assays.

### Quantification of lipids in tissues

Lipids were extracted from the liver, heart, kidney and WATepi according to the method by Bligh and Dyer^(29)^. The lipid extracts from liver, kidney and heart were evaporated to dryness under nitrogen and re-dissolved in isopropanol before quantification of TC and TAG on the Cobas c111 system using the CHOL2 (Cholesterol Gen.2) and TAG kits from Roche Diagnostics. The lipid extracts from WATepi were applied on silica gel plates (250 um Silica gel 60 from Merck KGaA) developed in hexane–diethyl ether–acetic acid (40:10:1, by vol)^(33)^ to separate the lipid classes as the high TAG content in WATepi would otherwise interfere with the enzymatic assay for cholesterol quantification. The free cholesterol and CE spots were identified using Rhodamine G (Fluka Chemie AG) and co-migration with known standards. The free cholesterol spot was scraped off and extracted twice with chloroform, evaporated to dryness and re-dissolved in isopropanol before quantification of TC using the CHOL2 (Cholesterol Gen.2) kit on the Cobas c111 system (Roche Diagnostics). The CE content in WATepi was too low for quantification.

### Cholesterol and bile acids in faeces

Lipids in faeces were extracted using methanol and chloroform, as described by Bligh and Dyer^(29)^. The lipid extracts were dried under nitrogen and re-dissolved in isopropanol, and the TC content was measured on the Cobas c111 system using the CHOL2 (Cholesterol Gen.2) kit from Roche Diagnostics. The faecal bile acid content was measured using the method described by Suckling *et al.* ^(34)^ using Chromabond C18 ec (3 ml/200 mg, Macherey-Nagel) and the Total Bile Acid Assay Kit (Diazyme Laboratories, Inc.) on the Cobas c111 system.

### Protein analyses in liver

Liver samples were homogenised in PBS. The tissue protein content was quantified using the Bradford dye-binding method^(35)^, with protein assay dye reagent (Bio-Rad Laboratories) and bovine serum albumin (Bio-Rad Protein Assay Standard II, Bio-Rad Laboratories) as the standard. Acetyl-CoA carboxylase (ACC) was measured using the Rat ACC ELISA Kit (Sandwich ELISA), cat no. LS-F35376 (LifeSpan BioSciences, Inc.). Cholesterol 7 *α*-hydroxylase (CYP7A1) was measured using the Rat CYP7A1 ELISA Kit (Sandwich ELISA), cat no. LS-F9946 (LifeSpan BioSciences, Inc.). Cluster of differentiation 36 (CD36, also known as fatty acid translocase) was measured using the Rat CD36 ELISA Kit (Sandwich ELISA), cat no. LS-F37404 (LifeSpan BioSciences, Inc.). Diacylglycerol O-acyltransferase 2 (DGAT2) was measured using the Rat DGAT2 ELISA Kit, cat no. EKX-MN0QK8 (Nordic Biosite). 3-Hydroxy-3-methylglutaryl CoA reductase was measured using the rat 3-Hydroxy-3-methylglutaryl CoA reductase/HMGCR ELISA Kit (Sandwich ELISA), cat no. LSF15758 (LifeSpan BioSciences, Inc.). LDL receptor was measured using the Rat LDLR/LDL Receptor ELISA Kit (Sandwich ELISA) cat no. LS-F11934 (LifeSpan BioSciences, Inc.). Microsomal TAG transfer protein (MTTP) was measured using the Rat MTTP/MTP ELISA Kit (Sandwich ELISA), cat no. LSF25381 (LifeSpan BioSciences, Inc.). Proprotein convertase subtilisin/kexin type 9 was measured using the Rat PCSK9 ELISA Kit (Sandwich ELISA), cat no. LS-F9168 (LifeSpan BioSciences, Inc.). Scavenger receptor class B, member 1 (SCARB-1) was measured using the Rat SCARB-1/SR-BI ELISA Kit (Sandwich ELISA), cat no. LS-F23258 (LifeSpan BioSciences, Inc.). Solute carrier family 2 (facilitated GLUT), member 2 (SLC2A2) was measured using the Rat SLC2A2/GLUT2 ELISA Kit (Sandwich ELISA), cat no. LSF25346 (LifeSpan BioSciences, Inc.). Sterol O-acyltransferase 2 (SOAT2) was measured using the Rat Sterol O-acyltransferase 2, SOAT2 ELISA Kit, cat no. MBS9336783 (MyBioSource Inc.).

Plates were read at SpectraMax Plus384 Microplate Reader (Molecular Devices) or Multiscan FC (Thermo Scientific), and all samples were analysed simultaneously in the same plate from each of the ELISA assays, with CVs < 5 %. The concentrations of the individual proteins are presented relative to total protein content.

### Outcome measurements

The primary outcome was to investigate if intake of diets added either a herring oil containing CA or a CA concentrate would affect the circulating TC concentration in obese Zucker *fa/fa* rats. The secondary aims were to investigate any effects of CA intake on a selection of hepatic enzymes and receptors involved in cholesterol metabolism, lipogenesis and VLDL assembly with the purpose to try to explain the mechanisms behind any cholesterol-lowering effect of CA intake in these rats.

### Statistical analyses

This study was primarily designed to investigate the effects of diets with HERO containing CA or a CA-rich concentrate on fatty acid composition in tissues and inflammatory responses in obese Zucker *fa/fa* rats^(26)^. Since this was the first study that tested the effect of CA with this outcome in obese Zucker *fa/fa* rats, no data on effect size were available for sample size calculation or minimally detectable effect sizes. Based on studies conducted in rats and mice using CA-rich fish oils with group sizes of six to twelve rodents/group^(16)^, and our recent study using ZDSD rats fed diets containing herring oil, anchovy oil or soyabean oil with six rats/group^(23,36)^, we designed this study with ten rats per experimental group. One rat in the HERO group developed diabetes and was consequently excluded from statistical analyses, and therefore, the statistical analyses were conducted with *n* 10 rats in the Control group, *n* 9 rats in the HERO group and *n* 10 rats in the CECO group.

The statistical analyses were conducted using SPSS Statistics version 29 (SPSS, Inc., IBM Company). All data were evaluated for normality using the Shapiro–Wilks test, revealing that all variables were normally distributed; therefore, one-way ANOVA was used to compare the experimental groups. The experimental groups were compared using one-way ANOVA, with post hoc test Fisher’s least significance test as recommended by Hayter^(37)^ for comparisons of three groups. The cut-off value for statistical significance was set at a probability of 0·05.

## Results

### Fatty acids and cholesterol in the diets

The HERO and CECO diets were designed with twice as much CA in the CECO diet compared with the HERO diet (1·40 *v*. 0·70 wt%, respectively, Table 2). The shorter *n*-11 MUFA, that is gadoleic acid (C20:1*n*-11) and 7-octadecenoic acid (C18:1*n*-11), were detected in the HERO diet, and gadoleic acid but not 7-octadecenoic acid was detected in the CECO diet. CA, gadoleic acid and 7-octadecenoic acid were not detected in the Control diet. The *n*-3 PUFA EPA, docosapentaenoic acid (DPA, C22:5*n*-3) and DHA were found in the HERO diet and in the CECO diet, but not in the Control diet. The amount of EPA was comparable between the HERO and the CECO diets (0·17 and 0·20 wt%, respectively), whereas the DHA content was markedly higher in the HERO diet compared with the CECO diet (0·18 and 0·04 wt%, respectively). The complete fatty acid compositions of the diets are presented in Hansen *et al.* 2025^(26)^.

**Table 2.** Contents of selected fatty acids and cholesterol in the diets Table 2 long description.

Per 100 g diet	Control diet	HERO diet	CECO diet
C18:1 *n*-11, g	< LOQ	0·04	< LOQ
C20:1 *n*-11, g	< LOQ	0·05	0·08
C22:1 *n*-11, g	< LOQ	0·70	1·40
C20:5 *n*-3, g	< LOQ	0·17	0·20
C22:5 *n*-3, g	< LOQ	0·02	0·01
C22:6 *n*-3, g	< LOQ	0·18	0·04
Cholesterol, mg	15·5	21·8	15·5

HERO: herring oil; CECO, cetoleic acid concentrate; LOQ: level of quantification.

The complete fatty acid compositions of the diets are presented in Hansen *et al.* 2025^(26)^.

The cholesterol content was low in the experimental diets (< 0·03 wt%), but was marginally higher in the HERO diet compared with the CECO diet and the Control diet, with no difference between the two latter (Table 2).

### Cholesterol metabolism, storage and excretion

**Figure 1.**
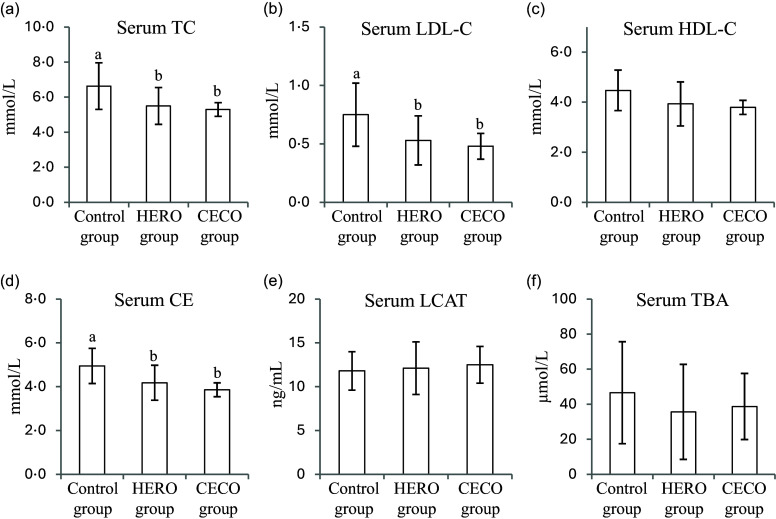
Serum concentrations of total cholesterol (TC) (a), LDL-cholesterol (b), HDL-cholesterol (c), cholesteryl esters (CE) (d), lecithin-cholesterol acyltransferase (LCAT) (e) and total bile acids (TBA) (f). Data are presented as mean and sd for *n* 10 in the Control group, *n* 9 in the HERO group and *n* 10 in the CECO group. Groups are compared using one-way ANOVA followed by Fisher’s least significance post hoc test when appropriate. Bars with different letters are significantly different (*P* < 0·05). HERO, herring oil; CECO, cetoleic acid concentrate.

The serum concentrations of TC (Figure 1(a)) and LDL-cholesterol (Figure 1(b)) were lower in the CECO and HERO groups compared with the Control group, with no differences between the HERO and CECO groups. The HDL-cholesterol concentration was similar in all groups (Figure 1(c)). Serum CE was lower in both HERO and CECO groups compared with Controls (Figure 1(d)), whereas the serum concentrations of lecithin-cholesterol acyltransferase (Figure 1(e)) and total bile acids (Figure 1(f)) were similar between all groups.

**Figure 2.**
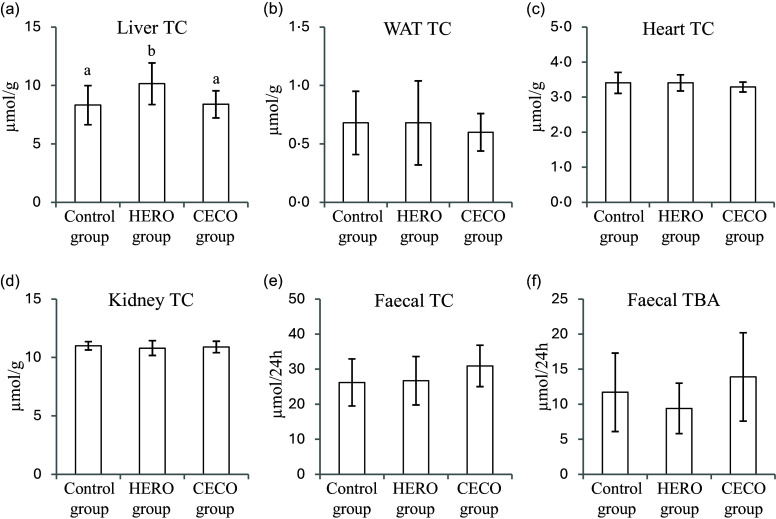
Total cholesterol (TC) content in liver (a), white adipose tissue (WAT) (b), heart (c) and kidneys (d), and 24-h faecal excretion of total cholesterol (e) and total bile acids (TBA) (f). Data are presented as mean and sd for *n* 10 in the Control group, *n* 9 in the HERO group and *n* 10 in the CECO group. Groups are compared using one-way ANOVA followed by Fisher’s least significance post hoc test when appropriate. Bars with different letters are significantly different (*P* < 0·05). HERO, herring oil; CECO, cetoleic acid concentrate.

The hepatic cholesterol content was higher in the HERO group compared with the Control group and the CECO group, with no differences between the CECO group and the Control group (Figure 2(a)). The amount of cholesterol found in WATepi (Figure 2(b)), heart (Figure 2(c)) and kidney (Figure 2(d)) were similar between all three groups. The faecal excretion of cholesterol (Figure 2(e)) and of total bile acids (Figure 2(f)) was also similar between all groups.

The liver concentrations (relative to protein) of 3-Hydroxy-3-methylglutaryl (HMG) CoA reductase (the rate-determining enzyme for the hepatic synthesis of cholesterol), LDL receptor, proprotein convertase subtilisin/kexin type 9 (secreted from the liver and binds to the LDL receptor thereby reducing the clearance of LDL), SCARB-1 (receptor for mature HDL) and CYP7A1 (the rate-determining enzyme for the conversion of cholesterol to bile acids) were similar between all groups (Table 3). The hepatic concentration of SOAT2 (catalysing the esterification of free cholesterol to CE for packaging into VLDL) was lower in both the HERO group and the CECO group when compared with the Control group.

**Table 3.** Liver enzymes and receptors related to cholesterol metabolism Table 3 long description.

ng/mg protein	Control group		HERO group		CECO group		*P* ANOVA
	Mean	sd	Mean	sd	Mean	sd	
HMG-CoA reductase	1574	635	1093	453	1269	348	0·12
LDL receptor	786	329	539	256	587	130	0·082
PCSK9	78·8	33·2	65·4	29·0	67·4	22·1	0·56
SCARB-1	1·31	0·57	0·93	0·35	1·02	0·28	0·13
CYP7A1	11·9	4·0	9·6	2·5	9·5	2·3	0·17
SOAT2	9·0	2·7^a^	6·7	1·7^b^	6·8	1·9^b^	0·040

HERO, herring oil; CECO, cetoleic acid concentrate; HMG-CoA, 3-Hydroxy-3-methylglutaryl CoA; PCSK9, proprotein convertase subtilisin/kexin type 9; SCARB-1, scavenger receptor class B, member 1; CYP7A1, cholesterol 7 *α*-hydroxylase; SOAT2, Sterol O-acyltransferase 2.

Data are presented as mean (sd) for *n* 10 in the Control group, *n* 9 in the HERO group and *n* 10 in the CECO group. Groups are compared using one-way ANOVA followed by Fisher’s least significance post hoc test when appropriate. Means in a row with different letters are significantly different. *P* < 0·05 was considered significant.

### Lipogenesis, lipid storage and VLDL secretion

**Figure 3.**
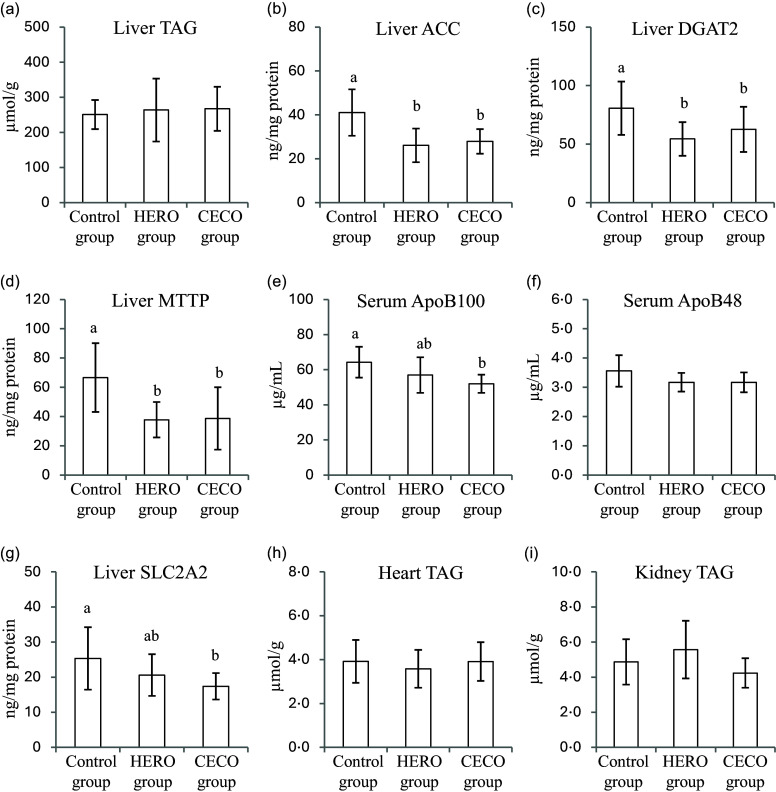
Liver contents of TAG (a), acetyl-CoA carboxylase (ACC) (b), diacylglycerol O-acyltransferase 2 (DGAT2) (c) and microsomal TAG transfer protein (MTTP) (d), serum concentrations of ApoB100 (e) and ApoB48 (f), liver solute carrier family 2 (facilitated glucose transporter), member 2 (SLC2A2) (g), and TAG contents in heart (h) and kidneys (i). Data are presented as mean and sd for *n* 10 in the Control group, *n* 9 in the HERO group and *n* 10 in the CECO group. Groups are compared using one-way ANOVA followed by Fisher’s least significance post hoc test when appropriate. Bars with different letters are significantly different (*P* < 0·05). HERO, herring oil; CECO, cetoleic acid concentrate.

The hepatic TAG concentration was similar between all groups (Figure 3(a)). The hepatic concentration of ACC (catalyses the carboxylation of acetyl-CoA to malonyl-CoA in the first and rate-limiting step in the biosynthesis of fatty acids, Figure 3(b)) and of DGAT2 (catalyses the final step in the TAG synthesis and regulates VLDL production, Figure 1(c)) was lower in the HERO group and the CECO group compared with the Control group. The lipidation of ApoB100 is catalysed by MTTP, and the hepatic concentration of MTTP was lower in both the HERO group and the CECO group compared with the Control group (Figure 3(d)). The serum ApoB100 concentration was lower in the CECO group compared with Controls, with no difference between HERO and Control groups and between HERO and CECO groups (Figure 3(e)), whereas the serum concentration of ApoB48 (Figure 3(f)) was similar between all groups. The hepatic concentration of SLC2A2, the major glucose transporter in the liver and an important provider of glucose as precursor for the formation of acetyl-CoA, was lower in the CECO group compared with the Control group (Figure 3(g)), with no difference between the HERO group and the Control group. The TAG contents in the heart (Figure 3(h)) and in the kidneys (Figure 3(i)) were similar between the groups.

**Figure 4.**
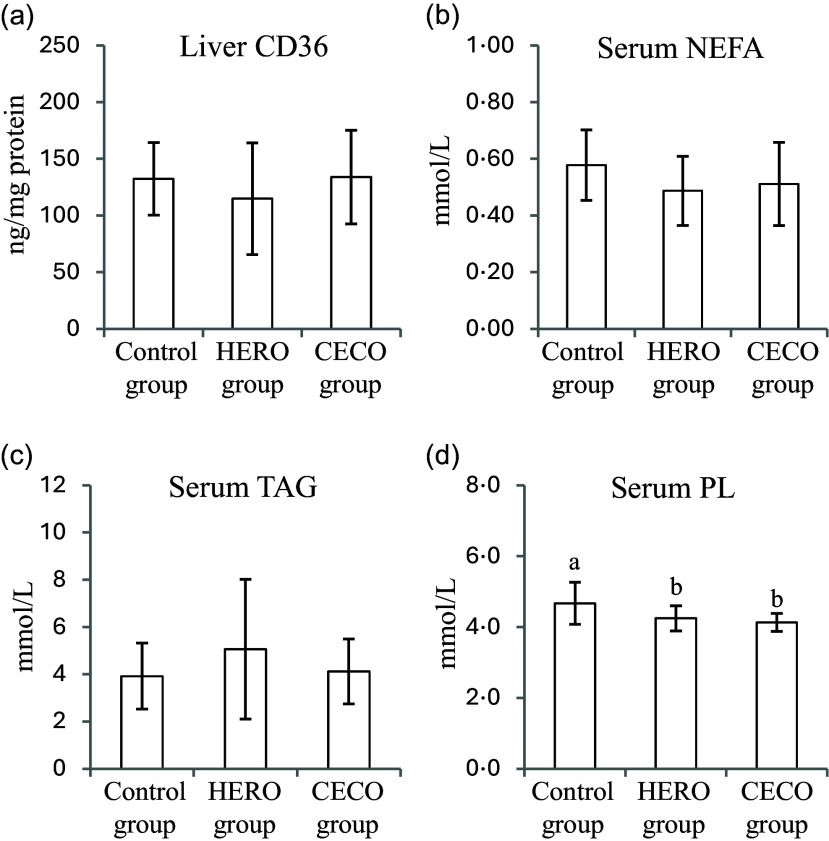
Liver content of cluster of differentiation 36 (CD36) (a), serum concentration of NEFA (b), serum concentration of TAG (c) and serum concentration of choline-containing phospholipids (PL) (d). Data are presented as mean and sd for *n* 10 in the Control group, *n* 9 in the HERO group and *n* 10 in the CECO group. Groups are compared using one-way ANOVA followed by Fisher’s least significance post hoc test when appropriate. Bars with different letters are significantly different (*P* < 0·05). HERO, herring oil; CECO, cetoleic acid concentrate.

The hepatic concentration of CD36 (a fatty acid translocase that facilitates the uptake of NEFA) was similar in all groups (Figure 4(a)). The serum concentrations of NEFA (Figure 4(b)) and TAG (Figure 4(c)) were also similar between the groups, whereas the serum concentration of choline-containing phospholipids was lower in the HERO and CECO groups compared with the Control group (Figure 4(d)).

## Discussion

The important and novel findings in the present study are firstly that consumption of diets containing herring oil or a CA concentrate produced from herring oil resulted in a lower serum TC concentration in obese hyperlipidaemic Zucker *fa/fa* rats, probably due to a combination of down-regulation of the *de novo* lipogenesis from glucose, lower TAG and CE syntheses and reduced lipidation of ApoB100, ultimately resulting in less cholesterol being exported from the liver in VLDL. Our analyses indicate that the lower serum TC concentration in the HERO and CECO groups was not a result of suppressed hepatic cholesterol synthesis, up-regulated removal of cholesterol by the liver, more cholesterol being stored in liver and extrahepatic tissues, or enhanced secretion of cholesterol or bile acids in faeces.

CA and its two chain-shortened metabolites gadoleic acid and 7-octadecenoic acid were recovered in liver from ZDSD rats^(36)^ and obese Zucker *fa/fa* rats^(26)^ fed herring oil and in obese Zucker *fa/fa* rats fed the CECO diet^(26)^, in amounts that reflected the relative contents of these *n*-11 MUFAs in the diets. Since the *n*-11 MUFAs are accumulated in the liver lipids, they may affect the hepatic metabolic pathways involved in lipid metabolism.

In a recent systematic review with meta-analyses, we presented evidence that the serum TC concentration was lower in rodents consuming diets with CA from fish oils or fish oil concentrates^(16)^. A subgroup meta-analysis revealed that this effect was evident regardless of whether the comparator groups consumed diets containing vegetable or animal sources of fat^(16)^. The systematically reviewed articles did not provide sufficient information to conclude how intake of CA may lead to lower circulating TC concentration^(16)^, and with the relatively wide spectrum of rodent strains and genetic mice models as well as differences in designs in these studies, it can be expected that different aspects of the cholesterol homeostasis may be affected by CA. A few suggestions for the mechanisms behind CA’s effect on cholesterol have been put forward; based on a lower hepatic mRNA level of HMG-CoA reductase in male C57BL/6J mice, it was suggested that a lower plasma TC could be a result of down-regulation of cholesterol synthesis in the liver after intake of a diet with pollock oil mixed with lard (estimated content of CA; 1·85 g/100 g diet^(16)^) compared with a diet containing lard^(38)^. Two other studies by the same research group reported highly induced hepatic mRNA level of CYP7A1 in female LDLr-/- mice and in male apoE-/ mice fed a diet containing a long-chain MUFA concentrate with milk fat and added cholesterol, without affecting the plasma TC concentration in either mouse model^(39)^. The estimated contents of CA were 0·70 and 1·81 g/100 g diet in the LDLr-/- mice and the apoE-/ mice experiments, respectively^(16)^. Although the faecal excretion of cholesterol or bile acids was not directly measured in the mouse experiments^(39)^, the up-regulation of CYP7A1 gene expression in liver indicates an elevated biosynthesis of bile acids from cholesterol in the liver and consequently higher faecal bile acid excretion. This corresponds well with the increased faecal output of bile acids seen in ZDSD rats experiencing a lower serum TC concentration after consuming a diet containing HERO (0·70 g CA/100 g diet), although the CYP7A1 protein content in liver was not affected^(23)^. In the present study, the lower serum TC concentration in the HERO and CECO groups probably cannot be explained by increased bile acid synthesis or lower reabsorption of bile in the enterohepatic cycle, since neither the hepatic protein content of CYP7A1, the serum total bile acid concentration nor the faecal excretion of bile acids were affected by these diets. Also, the endogenous cholesterol synthesis was probably not down-regulated after HERO or CECO intake, since the hepatic HMG-CoA reductase concentration was similar to that of the Control group.

The circulating TC concentration is regulated in several ways, including regulation of uptake to the liver and extrahepatic tissues through binding to receptors such as LDL receptor and SCARB-1 on the cell surface. In the present study, neither of these receptors were significantly affected by the CECO diet. The hepatic proprotein convertase subtilisin/kexin type 9 concentration was also similar between the groups, further supporting that the lower serum TC in the CECO group was not a result of increased uptake of cholesterol by the LDL receptor in the liver. Also, the cholesterol content in liver, WATepi, heart and kidney was similar between the CECO and Control groups, and together, these findings indicate that the lower serum TC after CECO intake was not a result of increased removal of cholesterol from circulation by hepatic or extrahepatic tissues.

TAG for VLDL assembly originates from NEFA bound to albumin in the circulation, *de novo* synthesis of fatty acids, and from uptake of chylomicron and VLDL remnants. CD36 is a fatty acid translocase and facilitates NEFA uptake to the liver. In the present study, neither the serum NEFA concentration nor the hepatic CD36 protein content was affected by the CECO diet, thus indicating that the uptake of NEFA from adipose tissue to the liver and the MTTP-regulated assembly of VLDL were probably not influenced by the CECO diet. To further attempt to explain the mechanisms of action behind the lower serum TC concentration in rats fed the CECO diet, we focused on VLDL since it is the main exporter of cholesterol from the liver. The secretion of VLDL is regulated by the amount of ApoB100 and lipids available to be packaged into the particle. The supply of TAG and CE for incorporation into VLDL is regulated by SLC2A2, ACC, DGAT2 and SOAT2, and the lipidation of ApoB100 is catalysed by MTTP. In the CECO group, the lower liver protein contents of SLC2A2, ACC, DGAT2, SOAT2 and MTTP suggest a lower hepatic synthesis of VLDL when compared with the Control group. This, together with the lower serum concentrations of ApoB100 and choline-containing phospholipids, strongly indicates that the VLDL secretion from the liver was down-regulated in the CECO group, and in consequence, less cholesterol was secreted into the circulation. A lower secretion of cholesterol as VLDL from the liver may thus, at least in part, explain the lower serum concentrations of TC and LDL-cholesterol in the rats after CECO intake.

A lower VLDL secretion rate after CECO intake could be expected to result in a lower serum TAG concentration; however, we found no differences in serum TAG concentration between the groups. This is likely explained by the abnormally slow clearance of chylomicrons in obese Zucker *fa/fa* rats^(21)^, resulting in a high circulating TAG concentration with a fatty acid composition that reflects that of the diet ^(40)^. The chylomicrons from obese Zucker *fa/fa* rats are characterised by a very high TAG content and a low content of cholesterol compared with VLDL, LDL and HDL^(41)^ and therefore have little impact on the serum TC concentration in these rats. The similar TAG contents in kidney and heart between the groups also indicate that the transport of TAG in chylomicrons and VLDL to these organs was not affected by the CECO diet. The rats in the present study were fasted for 6 h before euthanisation, and as expected, the serum TAG concentration was high in all rats, with no difference in ApoB48 between the dietary groups. It is therefore likely that the high chylomicron concentration concealed any changes in the VLDL-TAG concentration resulting from down-regulation of VLDL secretion in the CECO groups when serum TAG was quantified.

The HERO diet contained half the amount of CA as the CECO diet, but still, most of the same effects on lipid metabolism in the liver were observed as for the CECO diet. The lower serum concentrations of TC and LDL-cholesterol in the HERO group were accompanied by lower liver protein concentrations of ACC, DGAT2, SOAT2 and MTTP, thus indicating lower lipogenesis and cholesterol-acyl esterification and subsequent lower availability of TAG and CE for the assembly of VLDL for secretion from the liver, which is further supported by the lower serum CE concentration. Since the hepatic concentrations of LDL receptor and SCARB-1 were not affected by the HERO diet, the higher cholesterol content in the liver after consumption of the HERO diet may be, at least in part, a consequence of the marginally higher dietary cholesterol content combined with a lower secretion of VLDL. We recently presented evidence that when diabetic ZDSD rats were fed a diet containing herring oil, the serum TC concentration was lower compared with its control group fed soyabean oil, whereas neither the serum concentration of LDL-cholesterol nor the hepatic protein contents of ACC, MTTP, DGAT2^(23)^ or SOAT2 (Gudbrandsen, unpublished data) were affected. This indicates that the *de novo* lipogenesis from glucose, the TAG synthesis, the cholesterol esterification and the VLDL assembly and secretion were more susceptible for dietary influence in non-diabetic, hypercholesterolaemic obese Zucker *fa/fa* rats with fatty liver compared with ZDSD rats with overt diabetes.

Several findings in the present study point towards down-regulated secretion of VLDL as the explanation for the lower serum TC and LDL-cholesterol concentrations in the HERO and CECO groups. Down-regulation of MTTP will reduce the secretion of VLDL, which is the precursor of LDL. It has been demonstrated that the use of MTTP inhibitor medication reduced the LDL-cholesterol concentration in patients with homozygous familial hypercholesterolaemia, but unfortunately this effect was accompanied by an increased serum alanine transaminase concentration and accumulation of fat in the liver after 4 weeks of treatment^(42)^. Despite the lower MTTP protein content in the liver of Zucker *fa/fa* rats fed the HERO diet or the CECO diet, no effect was observed on the serum alanine transaminase concentration^(26)^ or the hepatic TAG content. A plausible explanation for this is that both diets also seem to reduce the *de novo* lipogenesis, the TAG synthesis as well as VLDL assembly and secretion, resulting in lower TC and LDL-cholesterol concentrations. Whether our findings have relevance for human biology remains to be investigated, since there are important differences in the cholesterol metabolism between rats and humans; whereas HDL is the main cholesterol transporter in rats^(43)^, humans transport most of the cholesterol in LDL^(44)^. This makes the present findings all the more interesting, since a reduction in LDL-cholesterol through down-regulation of MTTP may have an even greater beneficial impact in humans than in rats.

The present study has some strengths and limitations. Strengths include measurements of several central enzymes and receptors relevant to the cholesterol metabolism, *de novo* lipogenesis and VLDL assembly, as well as quantification of cholesterol in a variety of tissues as well as in faeces. Limitations to the study include the choice of animal model; we used obese Zucker *fa/fa* rats which have hyperlipidaemia and high activities of *de novo* lipogenesis and VLDL secretion. Although the obese Zucker *fa/fa* rat is a good model of human obesity and health-related aspects of obesity^(22)^, there are still substantial differences between rats and humans that limit the reliability and translation of the present findings to humans. It is, therefore, important to acknowledge that the observed effects in the current study could be specific for the obese Zucker *fa/fa* rat. The cholesterol content was low in the experimental diets, but was marginally higher in the HERO diet compared with the Control and CECO diets. A direct measurement of VLDL secretion was not conducted, which could have revealed if the lower serum TC concentration after intake of the HERO or CECO diets is a reflection of down-regulated VLDL secretion from the liver. Also, we cannot conclude that the observed effects of HERO or CECO intake are caused by CA alone, since both the herring oil and the CA concentrate contain a plethora of fatty acids with different chain lengths and degrees of unsaturation.

### Conclusion

In this study, we present evidence that consuming herring oil or a CA concentrate lowers the TC concentration in serum, probably as a result of down-regulation of VLDL secretion in response to lower lipogenesis. Neither the HERO diet nor the CECO diet seem to affect the hepatic cholesterol synthesis, the hepatic uptake of cholesterol from the circulation or the faecal excretion of cholesterol and bile acids.

## Table 1 Long description

A table comparing the compositions of three experimental diets: Control diet, HERO diet, and CECO diet. The table has 18 rows and 4 columns. The columns are labeled 'Contents (g/100 g diet)', 'Control diet', 'HERO diet', and 'CECO diet'. The rows list various contents and their respective amounts in grams per 100 grams of diet. Row 1: Soyabean oil, Control diet: 10.00, HERO diet: 6.30, CECO diet: 5.75. Row 2: Herring oil, Control diet: blank, HERO diet: 3.70, CECO diet: blank. Row 3: Cetoleic acid concentrate, Control diet: blank, HERO diet: blank, CECO diet: 4.25. Row 4: Casein, Control diet: 21.56, HERO diet: 21.56, CECO diet: 21.56. Row 5: Sucrose, Control diet: 9.00, HERO diet: 9.00, CECO diet: 9.00. Row 6: Cornstarch, Control diet: 48.23, HERO diet: 48.23, CECO diet: 48.23. Row 7: Cellulose, Control diet: 5.00, HERO diet: 5.00, CECO diet: 5.00. Row 8: t-Butylhydroquinone, Control diet: 0.0014, HERO diet: 0.0014, CECO diet: 0.0014. Row 9: Mineral Mix (AIN-93-MX), Control diet: 3.50, HERO diet: 3.50, CECO diet: 3.50. Row 10: Vitamin Mix (AIN-93-VX), Control diet: 1.00, HERO diet: 1.00, CECO diet: 1.00. Row 11: Growth and maintenance supplement, Control diet: 1.00, HERO diet: 1.00, CECO diet: 1.00. Row 12: L-Methionine, Control diet: 0.16, HERO diet: 0.16, CECO diet: 0.16. Row 13: L-Cystine, Control diet: 0.30, HERO diet: 0.30, CECO diet: 0.30. Row 14: Choline Bitartrate, Control diet: 0.25, HERO diet: 0.25, CECO diet: 0.25.

Navigate back to Table 1.

## Table 2 Long description

A table comparing the contents of selected fatty acids and cholesterol in three different diets: Control diet, HERO diet, and CECO diet. The table has 7 rows and 4 columns. The columns are labeled 'Per 100 g diet', 'Control diet', 'HERO diet', and 'CECO diet'. The rows are labeled with different fatty acids and cholesterol. Row 1: C18:1 n-11, g. Row 2: C20:1 n-11, g. Row 3: C22:1 n-11, g. Row 4: C20:5 n-3, g. Row 5: C22:5 n-3, g. Row 6: C22:6 n-3, g. Row 7: Cholesterol, mg. The values in the table are as follows: Row 1: Control diet < LOQ, HERO diet 0.04, CECO diet < LOQ. Row 2: Control diet < LOQ, HERO diet 0.05, CECO diet 0.08. Row 3: Control diet < LOQ, HERO diet 0.70, CECO diet 1.40. Row 4: Control diet < LOQ, HERO diet 0.17, CECO diet 0.20. Row 5: Control diet < LOQ, HERO diet 0.02, CECO diet 0.01. Row 6: Control diet < LOQ, HERO diet 0.18, CECO diet 0.04. Row 7: Control diet 15.5, HERO diet 21.8, CECO diet 15.5.

Navigate back to Table 2.

## Table 3 Long description

The table presents liver concentrations of various enzymes and receptors related to cholesterol metabolism across three groups: Control group, HERO group, and CECO group. The table has 6 rows and 8 columns. The columns are labeled as follows: ng/mg protein, Control group Mean, Control group SD, HERO group Mean, HERO group SD, CECO group Mean, CECO group SD, and P ANOVA. The rows are labeled with the names of different enzymes and receptors: HMG-CoA reductase, LDL receptor, PCSK9, SCARB-1, CYP7A1, and SOAT2. Each row provides the mean and standard deviation (SD) values for each group, along with the P value from ANOVA analysis. Row 1: HMG-CoA reductase, Control group Mean 1574, Control group SD 635, HERO group Mean 1093, HERO group SD 453, CECO group Mean 1269, CECO group SD 348, P ANOVA 0.12. Row 2: LDL receptor, Control group Mean 786, Control group SD 329, HERO group Mean 539, HERO group SD 256, CECO group Mean 587, CECO group SD 130, P ANOVA 0.082. Row 3: PCSK9, Control group Mean 78.8, Control group SD 33.2, HERO group Mean 65.4, HERO group SD 29.0, CECO group Mean 67.4, CECO group SD 22.1, P ANOVA 0.56. Row 4: SCARB-1, Control group Mean 1.31, Control group SD 0.57, HERO group Mean 0.93, HERO group SD 0.35, CECO group Mean 1.02, CECO group SD 0.28, P ANOVA 0.13. Row 5: CYP7A1, Control group Mean 11.9, Control group SD 4.0, HERO group Mean 9.6, HERO group SD 2.5, CECO group Mean 9.5, CECO group SD 2.3, P ANOVA 0.17. Row 6: SOAT2, Control group Mean 9.0, Control group SD 2.7, HERO group Mean 6.7, HERO group SD 1.7, CECO group Mean 6.8, CECO group SD 1.9, P ANOVA 0.040.

Navigate back to Table 3.
